# Vulvar and Vaginal Graft-Versus-Host Disease: prevalence and repercussions on vaginal microbiota

**DOI:** 10.61622/rbgo/2025rbgo471

**Published:** 2025-10-21

**Authors:** Luiza de Amorim de Carvalho Araújo, Karinne Cisne Fernandes Rebouças, Fernando Barroso Duarte, Eleutério José, Raquel Autran Coelho Peixoto

**Affiliations:** 1 Universidade Federal do Ceará Maternidade Escola Assis Chateaubriand Fortaleza CE Brazil Maternidade Escola Assis Chateaubriand, Universidade Federal do Ceará, Fortaleza, CE, Brazil.; 2 Universidade Federal do Ceará Departamento de Cirurgia Fortaleza CE Brazil Departamento de Cirurgia, Universidade Federal do Ceará, Fortaleza, CE, Brazil.; 3 Universidade Federal do Ceará da Criança e do Adolescente Departamento de Saúde da Mulher Fortaleza CE Brazil Departamento de Saúde da Mulher, da Criança e do Adolescente, Universidade Federal do Ceará, Fortaleza, CE, Brazil.

**Keywords:** Graft vs host disease, Vaginal diseases, Vagina, Atrophy, Dyspareunia, Bone marrow transplantation, Hematopoietic stem cell transplantation

## Abstract

**Objective:**

Allogeneic bone marrow transplantation (BMT) carries the risk of the donor's cells recognizing the recipient as abnormal, triggering Graft Versus Host Disease (GVHD). This study aimed to understand the prevalence and gynecological complications, including vaginal microbiota evaluation.

**Methods:**

A descriptive cross-sectional study was carried out from December 2022 to October 2023. Post-allogeneic BMT patients underwent gynecological evaluation at the Assis Chateaubriand Maternity School in Fortaleza-Ceará, Brazil, composed of structured interview and physical examination, which included collection of fresh examination of vaginal content, gram bacterioscopy and cervicovaginal cytology.

**Results:**

A total of 22 patients between 21 and 61 years old (average: 38) were evaluated, with an average of 1028 (± 979) days post BMT. Of these patients, whether or not they had Vulvar and Vaginal Graft Versus Host Disease (VVGVHD), 15 reported various gynecological complaints (dryness being the most common). Of the total, twelve showed signs of genital atrophy on examination. A 45% prevalence of VVGVHD was found, with vulvar and vaginal involvement of 100% and 60%, respectively. Burning and dyspareunia symptoms were more prevalent in patients with VVGVHD than in those without it (p<0.05); there was no difference in Human Papillomavirus (HPV) induced lesions between the two groups. However, compared to the population not undergoing allogeneic BMT, these patients had a higher prevalence of induced HPV lesions and intermediate vaginal flora.

**Conclusion:**

The findings described in the present work are consistent with other studies available in recent literature. In conclusion, VVGVHD is a potentially mutilating condition, with a significant prevalence among post-BMT patients.

## Introduction

There are several complications that affect patients after allogeneic bone marrow transplantation (BMT), among which infections and Graft Versus Host Disease (GVHD) are particularly noteworthy due to their frequency and severity.^(1-3)^ Around 30 to 50% of patients undergoing allogeneic BMT can develop GVHD, reaching a mortality rate of approximately 20%.^(4)^ The sites and organs affected are diverse, and GVHD can affect the skin, eyes, oral mucosa, liver, intestines as well as the genital region in a single or associated form.^(2,5)^ In female patients, vulvar and vaginal involvement can occur, which constitutes a specific subtype of GVHD, vulvar and vaginal GVHD (VVGVHD), first reported in 1982, based on the surgical management of 5 patients with late findings.^(6)^ The cutoff point traditionally accepted and used to differentiate acute and chronic GVHD is the 100th day after transplantation, with acute manifestations (GVHDa) commonly affecting the epithelium of various organs and structures and the chronic condition (GVHDc) associated with fibrosis and structural scarring alterations of such structure.^(7)^

In a recent review, 10 studies were reported that sought to clarify the incidence and prevalence of VVGVHD manifestations, with heterogeneous results with prevalence ranging from 5.9% to 88%.^(8)^ It is worth noting that possibly the varied designs of the studies reported, different diagnostic criteria used, multiple follow-up intervals and overlapping symptoms with genital atrophy associated with premature ovarian failure (POI) may explain the wide variation in incidence and prevalence reported in recent literature.^(2,9,10)^

The clinical manifestations of VVGVHD are varied and can present in both adult and pediatric patients.^(11,12)^ The vulva is most commonly affected and, generally, when there is vaginal involvement, there is a previous vulvar manifestation, isolated vaginal involvement being rare and potentially related to less symptomatic mutilating cicatricial changes when compared to vulvar involvement.^(13,14)^ Several symptoms may be present, including superficial and deep dyspareunia; postcoital bleeding; burning, itching, pain and other vulvar and general dysesthesias; vaginal discharge; dryness and dysuria; which must be differentiated from genital atrophy, also common in such patients.^(13,14)^ On vulvar inspection, there may be ulcerations, erosions, architectural loss (such as erasure or fusion of the labia minora; difficulty in exposing the clitoral glans due to atrophy and incarceration of the hood), fissures and pale striae.^(5,14,15)^ In the vaginal mucosa, dryness, enanthem, pallor, erosion and petechiae may be present.^(8)^ In its chronic version, there may be progression of synechiae culminating in vaginal stenosis, with formation of hematocolpus or hematometra, as well as potential impairment of sexual life, due to dyspareunia and difficulty or even impossibility of penetrative activity.^(16,17)^ It may be difficult to access the cervix to collect cervicovaginal cytology, making it difficult to screen for high-grade vaginal and cervical lesions.^(18,19)^

There are few recent studies that evaluate the prevalence and clinical course of VVGVHD and its nuances in the Brazilian context. It is hypothesized that, similarly to what is described in international literature, there is a population of patients who are underdiagnosed and progressing to various negative outcomes with disastrous repercussions on their quality of life. It is also possible that such patients, due to the particularities already described, are more vulnerable to genital infectious changes, which is aggravated by the commonly associated periods of post-BMT immunosuppression.^(20)^ In this sense, the present study aims to evaluate these patients after allogeneic BMT in order to characterize genital involvement and gynecological complications in this population, highlighting the possible diagnostic criteria for VVGVHD and various associations.

## Methods

A cross-sectional descriptive observational study was carried out in the Gynecology outpatient clinics of Assis Chateaubriand Maternity School in Fortaleza-Ceará, Brazil, from December 2022 to October 2023, including female patients post-allogeneic marrow transplant, over 18 years of age, non-pregnant, by opportunistic sampling. Socioeconomic characteristics were assessed (age, education, marital status, ethnicity, sexual orientation); data related to the transplant and its complications (allogeneic transplant from a related or unrelated donor, transplant time, extragenital GVHD manifestations, use of previous and current immunosuppressive medications); local symptoms reported by the patient, prior to BMT, post-BMT and current symptoms perceived at the time of evaluation (burning, itching, dryness, pain, dyspareunia, among others); signs of genital atrophy, assessment of the presence and degree of involvement related to VVGVHD, according to the classification proposed by the National Institutes of Health (NIH) in 2014 and Stratton et.al in 2007; cytological changes in the cervix; characterization of bacterioscopy, changes present in the fresh examination and colposcopy evaluation.^(7,15)^ Data will be collected using the tools described. Since the prevalence of VVGVHD in its different stages is a quantitative variable with probable normal distribution, Student's T-test was used, as well as chi-square or Fisher's exact test to evaluate qualitative correlations, with a p value <0.05 being considered statistically significant. The research protocol was approved by the institutional research and ethics committee.

## Results

A total of 22 patients between 21 and 61 years old (with a measured age of 38 years) undergoing allogeneic BMT were evaluated, with an average of 1028 days post-BMT (from 90 to 2920 days), indicated by different hematological diseases, as shown in the table. The majority were mixed race, had completed high school and underwent BMT as a relative of their sister. All declared themselves heterosexual (table 1).

**Table 1:** Clinical-demographic data of the participants (n=22)

Variable		n(%)
Age		38 ± 11(38)*
Ethnicity		
	White	4(18)
	Brown	1(77)
	Black	1(4.5)
Schooling		
	Illiterate	1(4.5)
	2nd degree complete	11(50)
	Incomplete 2nd degree	3(14)
	3rd degree complete	5(23)
	Incomplete 3rd degree	2(9.1)
Sexual orientation		
	Heterosexual	22(100)
Allogeneic BMT subtype		
	Related (brother)	4(18)
	Related (sister)	13(59)
	Unrelated	5(23)
Basic disease		
	Aplastic anemia	2(9.1)
	Lymphoma	2(9.1)
	Acute lymphoid leukemia	3(14)
	Acute lymphoid leukemia B	1(4.5)
	Acute myeloid leukemia	9(41)
	Chronic myeloid leukemia	2(9.1)
	Myelodysplastic syndrome	3(14)

* Mean ± SD (Median)

Numerous gynecological complaints were reported prior to the evaluation and that appeared post-BMT by seven patients (burning, dyspareunia, pain, dryness, formation of ulcers and itching), however, when current complaints were evaluated, 15 patients reported having some complaint, being dryness (13), burning (7) and dyspareunia (7) are the most common, in addition to pain (3), itching (2), sinusirrhage (2) and local edema (1).

A total of 3 (14%) patients had a fresh examination compatible with the presence of pseudohyphae, spores and/or yeast; 1 (4.5%) presented an evaluation compatible with bacterial vaginosis. Assessed using the Nugent score, the microbiota of 15 patients (68%) resulted in intermediate vaginal flora (score of 4 to 6), with only 3 (14%) of the tests described as normal vaginal flora and 2 (9.1%) of them presented pathological flora, confirming bacterial vaginosis using the method. It is important to highlight that none of the patients reported complaints of significant discharge during the initial anamnesis and that two of the samples collected presented little material for reading.

On cervicovaginal cytology, 18% of patients showed squamous cell atypia and high-grade lesions could not be ruled out (ASC-H); 18%, squamous cell atypia of undetermined significance (ASC-US); 9.1% low-grade intraepithelial lesion (LSIL); and 65% showed no changes.

Of the 22 patients evaluated, ten patients had signs of VVGVHD (prevalence of 45%), one of them had an extensive vaginal lesion (whose biopsy revealed to be a high-grade vaginal intraepithelial neoplasia lesion), another had a lesion on the cervix compatible with LSIL (having received appropriate treatment) and 12 patients showed signs compatible with genital atrophy, which was not correlated with age when a cut point of 50 years old was used in comparison. None of the patients evaluated was diagnosed with vulvodynia or vaginismus.

The presence of extragenital GVHD was not presented as a possible factor associated with the presence of VVGVHD, being equally distributed between the groups of carriers and non-carriers. Similarly, the use of various immunosuppressants (such as corticosteroids, cyclophosphamide, among others), despite being frequent among patients (used by 14 of the 22 patients), was not correlated with the presence of VVGVHD at the time of evaluation, when comparing carrier and non-carrier patients of VVGVHD (table 2).

**Table 2:** Comparison of clinical and demographic variables between VVGVHD and non-VVGVHD carriers (n=22)

Variable		Yes n(%)	No n(%)	p-value
Total		n=10	n=12	
Age		34 ± 11(31)	42 ± 10(42)	0.09
Ethnicity				0.59
	White	1(10)	3(25)	
	Black	1(10)	0(0)	
	Curtain	8(80)	9(75)	
Schooling				0.28
	2nd degree complete	4(40)	7(58)	
	Incomplete 2nd degree	2(20)	1(8.3)	
	3rd degree complete	1(10)	4(33)	
	Incomplete 3rd degree	2(20)	0(0)	
	Illiterate	1(10)	0(0)	
Sexual orientation				
	Heterossexual	10(100)	12(100)	
Type of allogeneic tx				0.25
	Sibling	1(10)	3(25)	
	Related - sister	5(50)	8(67)	
	Unrelated	4(40)	1(8.3)	
Time in days		967 ± 935(548)*	1.079 ± 1.053(730)*	0.84
Non-genital GVHD		8(80)	5(42)	0.09
Extragenital GVHD absent		8(80)	6(50)	0.20
Symptoms				
	Burning	6(60)	1(8.3)	0.02
	Dyspareunia	6(60)	1(8.3)	0.02
	Pain	3(30)	0(0)	0.08
	Dryness	7(70)	6(50)	0.41
	Ulcers	0(0)	0(0)	
	Itch	2(20)	0(0)	0.19
	Sinusirrhage	2(20)	0(0)	0.19
	Edema	1(10)	0(0)	0.45
	Asymptomatic	2(20)	5(42)	0.38

* Mean ± SD (Median)

The diagnosis was made around 967 days (average) in the evaluation carried out in the present study (only 2 patients were evaluated and diagnosed with GVHDa, below 100 days of BMT) (table 03). Two of these patients were asymptomatic at the time of evaluation and the others had different gynecological complaints: burning (in 60% of patients), dyspareunia (60%), pain (30%), dryness (70%), itching (20%), sinusirrhage, (20%) and edema (20%).

**Table 3:** Severity scores VVGVHD according to Jagasia, 2015 and Stratton 2007 (n=10)

Evaluation score		n(%)
Jagasia et al. (2015) ^(7)^		
	1	2(9.1)
	2	5(23)
	3	3(14)
Stratton et al, 2007^(15)^		
	1	2(9.1)
	2	5(23)
	3	3(14)

**Figure 1:**
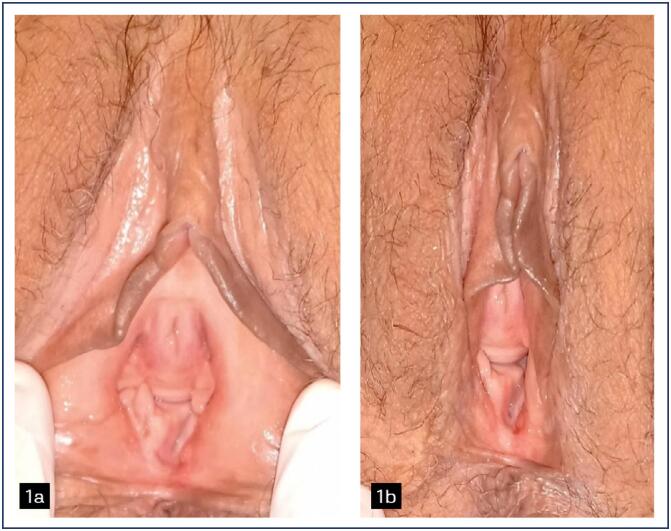
Grade I acute GVVV. 1a generalized pallor. 1b white, erythematous spots

**Figure 2:**
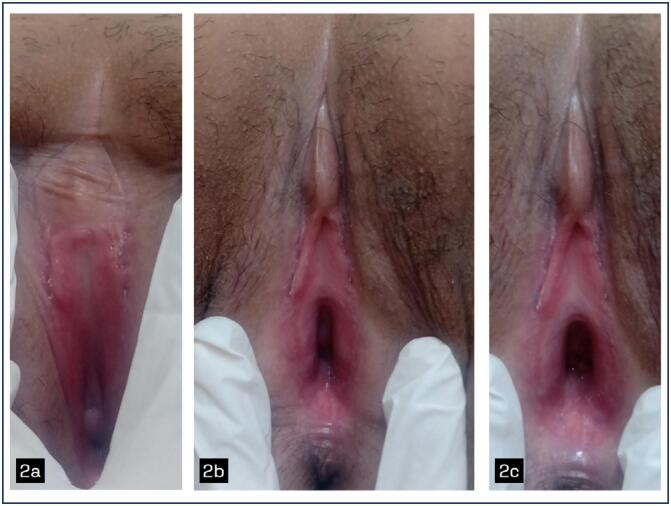
Grade III chronic VGVHD. 2a Lichen sclerosus-like: pallor, white sclerotic patches, atrophy, loss of labia minora. 2b Lichen planus-like: erythematous spots, generalized erythema. 2c Fibrotic changes: vulvar synechiae including clitoral capping/agglutination

**Figure 3:**
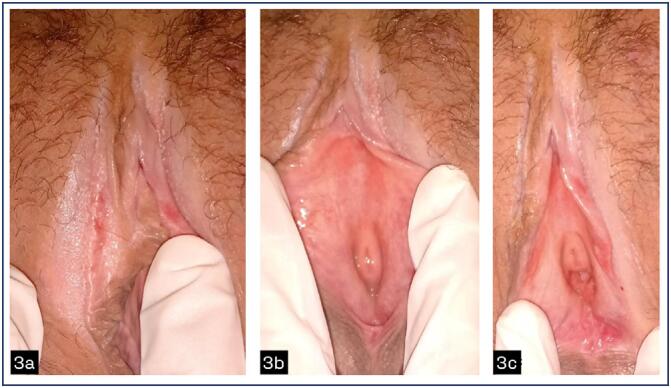
Chronic VGVHD grade II .3a white spot and erythematous spot, agglutination of labia minora. 3b Pallor and erythema. 3c Erosion of the furcula

**Figure 4:**
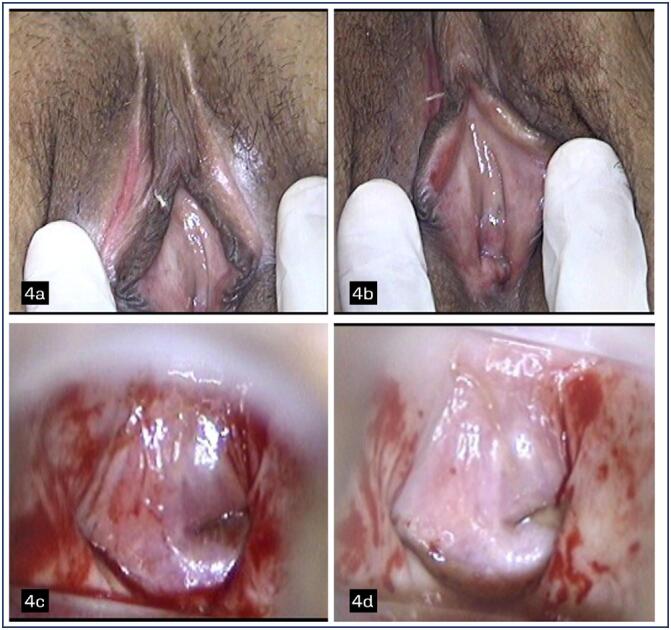
GVVVHD grade II. 4th discrete agglutination of the labrum minora on the right. 4b white spot and erythematous spot. 4C-D Vaginal involvement, fibrous ring in the upper third with difficulty in exposing the uterine place, in its entirety)

Vulvar involvement was present in all patients evaluated and vaginal involvement (with synechiae, middle and upper third stenosis) was present in 6 patients, 60% of the patients evaluated and at genital colposcopy, several local changes were noted (Figures 1 to 4).

The changes arising from cervicovaginal cytology, fresh examination and evaluation by Gram bacterioscopy with resulting Nugent score were distributed in an equivalent way and when comparing the groups of carriers and non-carriers of VVGVHD, without statistically significant impact, as well as the prevalence of signs compatible with genital atrophy associated or not with VVGVHD.

It is important to highlight that the absence of initially reported symptoms was not correlated with the degree of vulvar and vaginal involvement by GVHD, therefore, even asymptomatic, there is potential architectural impairment.

## Discussion

The prevalence of VVGVHD found in this study was 45%, compatible with a recent systematic review, which cites a prevalence between 1.6% and 69%, maintaining the prevalence range of other reviews of 5.9% to 88% and the prevalence cited in a national reference 24.9% to 69%.^(8,21,22)^ The diagnosis was made around 967 days (average) in the evaluation carried out in the present study (only 2 patients were evaluated and diagnosed with GVHDa, below 100 days of BMT), which is also found in the literature consulted, with diagnosis prevailing changes chronic and being carried out between 7 and 10 months after the BMT.^(22)^

It is important to note that all patients had vulvar involvement and 60% of them had vaginal involvement (either with erosions, thin synechiae or extensive fibrous rings with significant involvement of the middle and upper third, making it difficult to reach the cervix), with no isolated vaginal conditions being diagnosed, with this association being higher than the reported prevalence of association of vulvar and vaginal conditions in other studies.^(21,22)^ The majority of patients evaluated had advanced disease scores - score II in 23% of them and score III in 14%, according to the NIH classification, 2014 - suggesting possible diagnostic delay possibly related to non-recognition of specific symptoms, confusion with common symptoms related to genital trophy as well as a possible lack of structured and specialized gynecological follow-up.^(7)^

Baseline disease, BMT subtype, patient's age as well as the sex of the related donor did not appear to influence factors associated with the development of VVGVHD, however, given the small number of patients evaluated, it is not possible to state that this influence does not actually exist. The presence of extragenital GVHD was not associated with the presence of VVGVHD. Such factors are variably described in the literature, sometimes associated with the development of VVGVHD, which expresses the important heterogeneity in studies and the effects of retrospective assessments carried out, requiring more long-term prospective studies to scrutinize the real influence of such factors on the throughout follow-up.^(8,21,22)^

It is important to highlight that the development of GVHDc does not seem to be limited to the first year post-BMT and its consequences with structural involvement seem to extend with the prolongation of survival and even patients with more than 2 years of transplantation presented manifestations compatible with GVHDc in the present study, highlighting the importance of diagnostic alert and long-term follow-up of such patients.^(8,21,23)^ It is noteworthy that even with the high prevalence of signs compatible with genital atrophy, equally distributed among carriers and non-carriers of VVGVHD, patients with VVGVHD seem to report more burning and dyspareunia than patients without the condition, these being the most frequently reported symptoms.

There was a higher prevalence of HPV-induced cervicovaginal cytology changes in post-BMT patients, potentially related to transient immunosuppression carried out, loss of gynecological follow-up due to the potential severe acute disease in addition to important vaginal symptoms that may hinder access to the cervix. Considering the general population, studies report 6.4% to 8.9% of abnormal pap smear results, while among post-BMT patients a rate of 35% was found, which are consistent with other studies internationally, that showed Pap smear were 13% and 11% in allogeneic and autologous recipients respectively, higher than in the general Taiwanese population (1.22%).^(24-26)^ There was no difference in cytopathological abnormalities between the two study groups.

The importance of specialized clinical monitoring with an experienced team in identifying such patients is highlighted here. Gynecological evaluation prior to BMT is recommended, with patient training regarding signs and symptoms of VVGVHD, genital atrophy, and premature ovarian failure, as well as guidance on reproductive planning and evaluation of induced HPV lesions as suggested by recent literature, including by the Brazilian Society of Bone Marrow Transplantation in its recent consensus of 2021.^(13,22)^ Similarly, frequent subsequent follow-up is indicated, with reassessments at intervals of approximately 3, 6, and 12 months after BMT, these periods being critical for the diagnosis of acute conditions (up to 100 days) as well as including the most frequent reported periods (between 6 and 10 months) for the diagnosis of chronic conditions - in addition to specialized support with collection of cervicovaginal cytology during the maintenance of immunosuppressant use; approach to climacteric symptoms after premature ovarian failure with prescription of adequate hormone replacement for eligible patients; as well as adequate sexuality support; with routine annual follow-up being suggested after the first year of BMT.^(1,2,13,27,28)^

Furthermore, the importance of early management after identifying cases of VVGVHD is highlighted, with topical estrogen therapy and consideration of early hormone replacement therapy being recommended in eligible patients.^(2,21)^ It is also important to highlight the role of the use of topical corticosteroids in both vulvar and vaginal involvement, with strict monitoring for possible adverse effects as well as disease progression, as well as the possibility of using other immunomodulators, such as topical tacrolimus as a second line of treatment.^(21)^ The use of vaginal dilators and surgical procedures to address possible fibrous rings and vaginal synechiae formed come into play in more advanced and refractory cases, and may be associated with the pelvic physiotherapy approach.^(29-32)^

Little is known about the vaginal microbiota of patients after allogeneic BMT, especially in the population with VVGVHD, and it is possible that given the multiple local aggressors developed (such as POI, transient immunosuppression, local inflammation), these patients may experience changes in the local vaginal microbiota, being more susceptible to recurrent vulvovaginitis and its repercussions, similar to what is suggested to occur in contexts of genital atrophy, immunosuppression and local immune-mediated diseases, such as vulvar lichen sclerosus.^(33)^ To assess the local microbiota, fresh examination and bacterioscopy with evaluation of the Nugent score are methods that are easy to access and perform, as well as low-cost and widely available, and can characterize the presence of various microorganisms and confirm, alone or together, several of the prevalent vulvovaginitis, such as candidiasis and bacterial vaginosis, being the choice for diagnostic evaluation in various protocols and therapeutic plans.^(33,34)^

There are few studies clarifying microbiota changes in immunosuppressed patients or even with various chronic diseases suggesting that changes to the vaginal flora without predominance of lactobacilli can make the patient in question vulnerable to various gynecological complications.^(21,35)^ The present study revealed intermediate flora using the Nugent score in the majority of patients evaluated, most of whom had a normal fresh examination, with no distinction between carriers and non-carriers of VVGVHD, suggesting that such patients may be more vulnerable to the development of vaginal dysbiosis. It is possible that these changes are related, for example, to genital atrophy, also found with a prominent prevalence in the patients studied, without distinguishing such prevalence between those with and without VVGVHD.

Despite the various gynecological complications described in the groups studied, none of the patients had a diagnosis of vulvodynia or vaginismus, despite the gynecological complications already described (including genital atrophy and VVGVHD).

The sample size imposes itself as a limitation for the study of such patients as well as the single cross-sectional assessment, making it impossible to measure the incidence of VVGVHD and its subclassifications. It is also important to highlight that the cross-sectional evaluation of a single group of patients did not allow for proposals with a therapeutic focus for such patients. Despite the limitations, the importance of the present work stands out for the pioneering descriptive characterization of patients from the reference center in the state of Ceara-Brazil, enhancing future prospective studies in such a population as well as instigating possible interventionist proposals for the prevention and treatment of related gynecological complications to the BMT, not just VVGVHD.

The findings described in the present work are consistent with other studies available in recent literature, despite the limitations already mentioned.^(21,22)^ It is hoped that it can serve as a descriptive basis for other studies that are capable of evaluating primary, secondary and even tertiary prevention strategies for VVGVHD as well as all the gynecological complications described, to which post-BMT patients seem to be more vulnerable.

Prospective studies with long-term follow-up are necessary to clarify the cumulative incidence and possible prevention and treatment strategies for such complications, as a way of deciding the best course of treatment for patients as well as the best approach to complications should they occur. Furthermore, additional studies to evaluate the vaginal microbiome of such patients may be useful to clarify the behavior of microbiota alterations, with the use of tools such as next-generation sequencing (metagenomics), which may better clarify microbial alterations in immunosuppressed women.

## Conclusion

There was a high prevalence of patients diagnosed with GVHD in the studied population. No significant associations were identified between sociodemographic factors or factors related to the underlying disease, BMT subtype, or presence of extragenital GVHD and the development of VVGVHD. There were no significant colposcopy findings or changes in vaginal microbiota and cervicovaginal cytology in women with VVGVHD.

The importance of the present study lies in characterizing post-BMT patients as well as being alert to the potential development of VVGVHD in the long term. VVGVHD is a potentially mutilating occurrence, with potential long-term development, possibly increasing its incidence in parallel with the increase in survival of post-BMT patients. Specialized and structured long-term follow-up, as well as the training of such patients regarding the particularities of post-BMT gynecological follow-up, are essential for improving care for this population.

## Data availability

: The authors did not make the data from this article available in repositories prior to submission.
